# Stroke patients’ knowledge, attitudes, and practices regarding home-based exercise and psychological rehabilitation programs

**DOI:** 10.3389/fmed.2025.1598489

**Published:** 2025-06-26

**Authors:** Tianjiao Dai, Ying Zou, Hongbo Li, Wei Xu, Jing Luo, Lezheng Wang, Qiyue Sun, Lei Shi, Shaoxuan Ma, Ye Liu

**Affiliations:** ^1^School of Sport Science, Beijing Sport University, Beijing, China; ^2^School of Rehabilitation, Jiangsu College of Nursing, Huai'an, China; ^3^Department of Rehabilitation, Jinhu County People’s Hospital, Huai’an, China; ^4^Department of Rehabilitation, Zhenjiang Hospital of Integrated Traditional Chinese and Western Medicine, Zhenjiang, China; ^5^College of Physical Education, Yangzhou University, Yangzhou, China

**Keywords:** knowledge, attitude, practice, stroke, patient, home-based exercise, psychological rehabilitation programs

## Abstract

**Background:**

Stroke rehabilitation encompasses crucial components essential for the recovery process. This study aimed to investigate the knowledge, attitudes, and practices (KAP) of stroke patients regarding home-based exercise and psychological rehabilitation programs aimed at enhancing their physical and mental well-being, as well as alleviating depressive symptoms.

**Methods:**

This study conducted a cross-sectional survey at 37 institutions from December 2023 to January 2024. The survey gathered demographic data and assessed KAP related to stroke rehabilitation through structured questionnaires.

**Results:**

A total of 499 valid questionnaires were included in this study. Of these participants, 312 (62.5%) were male and 252 (50.5%) were aged over 60 years. The median [25.75%] knowledge, attitude, and practice scores were 9.03 (5.04) (possible range: 0–20), 33.19 (3.55) (possible range: 9–45), and 21.43 (4.49) (possible range: 6–30), respectively. Multivariate logistic regression showed that knowledge score (OR = 1.19, 95% CI: [1.13, 1.26], *p* < 0.001), attitude score (OR = 1.28, 95% CI: [1.19, 1.38], p < 0.001), employed (OR = 2.41, 95% CI: [1.15, 5.04], *p* = 0.020), retired (OR = 2.20, 95% CI: [1.20, 4.02], *p* = 0.011), and more than 3 years of duration of stroke (OR = 0.37, 95% CI: [0.16, 0.85], *p* = 0.019) were independently associated with practice. Structural equation modeling (SEM) showed that knowledge directly affected attitude (*β* = 0.407, *p* < 0.001), knowledge (*β* = 0.390, *p* < 0.001) and attitude (*β* = 0.461, *p* < 0.001) directly affected practice, and knowledge indirectly affected practice through attitude (*β* = 0.188, *p* = 0.007).

**Conclusion:**

The study highlights that while stroke patients generally exhibit positive attitudes and practices toward home-based exercise and psychological rehabilitation, there remains a gap in their knowledge about these crucial interventions. Clinically, it is imperative to enhance educational strategies targeting stroke survivors, focusing on improving knowledge to further strengthen their rehabilitation outcomes and adherence to prescribed regimens.

## Introduction

China, as the largest developing country in the world, is currently facing significant challenges in addressing the needs of its rapidly aging population. The average age of prevalent stroke patients in China is 66.4 years (1). Stroke, comprising ischemic and hemorrhagic types, is not only the second leading cause of death worldwide but also the foremost cause of mortality in China, home to the highest stroke burden globally (2–4). In individuals over 40, the standardized prevalence of stroke in China is 2.61%, with an incidence rate of 505.23 per 100,000 individuals, totaling approximately 17.8 million stroke patients (5). Furthermore, studies highlight significant psychosocial challenges faced by stroke patients, including prevalent anxiety and depression, experienced by 23 and 19% of patients, respectively, within the first 6 months post-stroke (6).

Effective stroke management primarily aims at reducing brain injury and enhancing patients’ recovery. The success of treatments heavily depends on the timely and accurate diagnosis by specialists, prompt initiation of treatment, and subsequent rehabilitation. Research indicates that the brain’s high plasticity allows for significant reductions in neurological deficits through immediate and long-term rehabilitation. However, the recovery process in post-stroke patients is notably varied due to factors such as the extent of the stroke, the degree of spontaneous regeneration, neuroplasticity, selected pharmacotherapy, and the adequacy of rehabilitation measures (7). Post-acute stroke rehabilitation typically incorporates two main models: Inpatient Rehabilitation (IR), which is provided within hospital settings, and Community Rehabilitation (CR), which includes outpatient services at clinics, day hospitals, or in the patient’s own home. Importably, the CR model features Early Supported Discharge (ESD), facilitating early hospital discharge with continued rehabilitation and support at home (8). Additionally, in China, there is a prevalent tradition of family-based rehabilitation, where family caregivers often take on the role of primary caregivers for stroke patients. This cultural practice underscores the integral role of familial support in the continuity of care and rehabilitation for stroke survivors.

The Knowledge, Attitude, and Practice (KAP) model is pivotal in public health, suggesting that individual behaviors are significantly influenced by one’s knowledge and attitudes. This approach is instrumental in examining health-related behaviors, where the assessment of knowledge and risk perception is frequently conducted through KAP surveys, shedding light on the intricate relationships between knowledge, attitudes, and behaviors (9–11). In China, which bears the heaviest global stroke burden and a significantly aging population, researching the KAP regarding Home-Based Exercise and Psychological Rehabilitation Programs among stroke patients is crucial. This study seeks to improve recovery outcomes and decrease morbidity by customizing interventions to the patients’ needs and the cultural framework, particularly focusing on the largely unexplored domain of family-supported home care predominant in China.

While a previous KAP study targeting family members of stroke survivors revealed relatively high scores in KAP questionnaires, it highlighted a gap in knowledge among caregivers in rural areas with lower education levels, making the patients more susceptible to potential complications of stroke (12). Despite this, there has been no similar research directly focusing on the patients. Therefore, this study aims to investigate the KAP of stroke patients concerning home-based exercise and psychological rehabilitation programs. The goal is to enhance their physical and mental well-being and alleviate symptoms of depression, which could lead to significant improvements in rehabilitation effectiveness and patient outcomes in this vital area.

## Materials and methods

### Study design and subjects

This cross-sectional study was conducted from December 2023 to January 2024 at 37 institutions and involved stroke patients as the research subjects. The study received approval from the Ethics Committee of Jinhu County People’s Hospital (LLSC 2023-LW-27), and all participants provided informed consent.

#### Inclusion criteria

Participants were included in the study if they:

1. Met the criteria set forth by the Fourth National Diagnostic Standards for Cerebrovascular Disease and were diagnosed with stroke through imaging tests such as CT or MRI.
2. Were in the subacute or recovery phase of stroke with stable conditions.
3. Possessed a sufficient level of education to understand the study procedures and could cooperate with the investigation.
4. Voluntarily agreed to participate in the study and signed the informed consent form.

#### Exclusion criteria

Individuals were excluded from the study if they:

1. Had Alzheimer’s disease.
2. Were diagnosed with malignant tumors, severe cardiovascular diseases, or significant liver and kidney dysfunction.
3. Had a long-term dependency on sedative drugs or were alcohol-dependent.
4. Had a family history of mental disorders.

### Questionnaire introduction

The questionnaire was meticulously designed with references to guidelines and relevant literature (13–16), incorporating input from five experts in clinical rehabilitation. The preliminary version underwent a small-scale pilot test (40 copies) to assess reliability, which was determined to be 0.914. The finalized version of the questionnaire was composed in Chinese and comprised four dimensions covering a total of 38 items: 12 items on basic information, 10 on knowledge, 9 on attitudes, and 7 on practices (See Supplementary questionnaire).

For scoring, the knowledge dimension used a three-point scale: “Very familiar” (2 points), “Have heard of it” (1 point), and “Unclear” (0 points), with total scores ranging from 0 to 20. Attitudes were measured on a five-point Likert scale; items A1-A4 and A6-A8 gaged positive attitudes and scored from 5 (strongly agree) to 1 (strongly disagree), whereas A5 and A9 assessed negative attitudes and scored inversely from 1 to 5, with the overall attitude score spanning 9 to 45 points. Practice items P1-P6 also utilized a five-point Likert scale, scoring from 5 (completely meets) to 1 (completely does not meet), with the score range for this dimension being 6 to 30. Item P7 was a multiple-choice question designed to identify the sources of current knowledge. A scoring threshold of over 70% was set for each dimension to classify adequate knowledge, positive attitudes, and proactive practices (17).

The questionnaire was made available electronically through the “Questionnaire Star” platform (Changsha Ranxing Information Technology Co., Ltd.). It was distributed in 37 tertiary and secondary specialty hospital departments across 13 cities in Jiangsu Province (See Supplementary Table S1) via QR codes in patients’ WeChat and QQ groups. Paper versions were also distributed in rehabilitation training halls and were filled out voluntarily. Assistance was provided to some patients who were unable to use mobile devices or handle writing tools, either by a family member or a therapist. A total of 106 responses were collected electronically, and 396 via paper. After corrections for suspected errors made through telephone or direct therapist contact, and the exclusion of two for incomplete responses, 500 valid questionnaires were retained. The data were compiled into an Excel spreadsheet and reviewed for completeness, consistency, and validity by the research team.

Sample size calculation:

The sample size for this cross-sectional study was determined using the standard formula for minimum sample size in prevalence studies (18):

$$n={\left(\frac{Z_{1}-\alpha /2}{\delta }\right)}^{2}\times p\times (1-p)$$

For this calculation:

The significance level (𝛼) is set at 0.05, giving a Z value of 1.96.

The margin of error (𝛿) is 0.05.

The estimated proportion (𝑝) is 0.5, assuming maximum variability.

Using these parameters, the required sample size (𝑛) is calculated to be 384. To account for a typical questionnaire response rate of 80%, the final sample size needed is adjusted to at least 480 questionnaires to ensure sufficient data for robust analysis.

### Statistical analysis

Data analysis was conducted using R 4.3.2 and Stata 18.0 (Stata Corporation, College Station, TX, USA). Continuous data are presented as means and standard deviations (SD), while categorical data are expressed as n (%). Continuous variables underwent a normality test, with the t-test for normally distributed data and the Wilcoxon Mann–Whitney test for non-normally distributed data when comparing two groups. For three or more groups with normally distributed continuous variables and uniform variance, ANOVA was used for comparisons, while the Kruskal-Wallis test was employed for non-normally distributed data. Univariate and multivariate logistic regression were performed to explore the risk factors associated with K, A, and P, with 70% of the total score was used as the cut-off value. Univariate variables with *p* < 0.05 were enrolled in multivariate regression. Structural equation modeling (SEM) was utilized to explore the relationships between knowledge (K), attitude (A), and practice (P). Model fit was evaluated using root mean square error of approximation (RMSEA), incremental fit index (IFI), Tucker–Lewis index (TLI), and comparative fit index (CFI). A two-sided *p*-value less than 0.05 was considered statistically significant.

## Result

In this study, we initially collected 502 questionnaires, from which we removed three due to irregularities: two from the attitude section and one from question 8 in the baseline section. This resulted in 499 valid questionnaires, achieving a validity rate of 99.40%. The internal consistency of both the total scale and its subscales was confirmed as being high, with a Cronbach’s alpha of 0.901 for the total scale, and 0.904, 0.804, and 0.836 for the knowledge, attitude, and practice sections, respectively.

Among the 499 stroke patients who participated in the study, 312 (62.5%) were male, 252 (50.5%) were over 60 years old, 249 (49.9%) lived in the city, 215 (43.1%) had junior high school education or below, 256 (51.3%) had monthly per capita income of less than 5,000 yuan, only 92 (18.5%) could take care of themselves, 301 (60.3%) had stroke for less than 1 year, 301 (60.3%) had exercise habit before onset, 448 (89.8%) were positive and optimistic before onset. The median [25.75%] knowledge, attitude, and practice scores were 9.03 (5.04) (possible range: 0–20), 33.19 (3.55) (possible range: 9–45), and 21.43 (4.49) (possible range: 6–30), respectively. Participants’ knowledge scores were more likely to vary depending on: residence (*p* < 0.001), education (*p* < 0.001), work status (*p* < 0.001), monthly per capita income (*p* < 0.001), marital status (*p* = 0.036), and whether have exercise habits before the onset of the disease (*p* < 0.001). Meanwhile, their attitude scores were more likely to vary across residence (*p* = 0.028), duration of stroke (*p* = 0.013), whether have exercise habits before the onset of the disease (*p* = 0.003), and whether positive and optimistic before the onset of the disease (*p* = 0.005). Furthermore, their practice scores were more likely to vary depending on: residence (*p* = 0.02), education (*p* = 0.011), work status (*p* = 0.03), whether have exercise habits before the onset of the disease (*p* < 0.001), and whether positive and optimistic before the onset of the disease (*p* = 0.046) (Table 1).

**Table 1:** Demographic characteristics.

*N* = 499	*N* (%)	Knowledge		Attitude		Practice	
		Median [25.75%] or mean (± SD)	*p*	Median [25.75%] or mean (± SD)	*p*	Median [25.75%] or mean (± SD)	*p*
Total score	499 (100.0)	9.03 (5.04)		33.19 (3.55)		21.43 (4.49)	
Gender			0.485		0.414		0.688
Male	312 (62.5)	9.15 (5.17)		33.12 (3.60)		21.39 (4.59)	
Female	187 (37.5)	8.84 (4.82)		33.29 (3.47)		21.49 (4.35)	
Age			0.922		0.445		0.958
18–44	73 (14.6)	9.21 (5.52)		33.58 (3.96)		21.52 (4.36)	
45–60	174 (34.9)	8.95 (4.71)		33.36 (3.43)		21.55 (4.04)	
>60	252 (50.5)	9.04 (5.13)		32.96 (3.50)		21.32 (4.83)	
Residence			<0.001		0.028		0.02
Rural area	197 (39.5)	7.86 (4.87)		32.77 (3.49)		20.86 (4.37)	
City	249 (49.9)	10.32 (4.98)		33.63 (3.36)		21.98 (4.47)	
Suburbs	53 (10.6)	7.34 (4.43)		32.62 (4.33)		20.92 (4.78)	
Education			<0.001		0.187		0.011
Junior high school and below	215 (43.1)	7.42 (4.73)		32.89 (3.46)		20.88 (4.81)	
High school/secondary vocational school	155 (31.1)	9.17 (4.84)		33.31 (3.74)		21.41 (4.16)	
College degree and above	129 (25.9)	11.56 (4.73)		33.53 (3.43)		22.36 (4.19)	
Work status			<0.001		0.856		0.03
Employed	101 (20.2)	10.06 (4.94)		33.39 (3.59)		22.11 (3.85)	
Unemployed	53 (10.6)	8.13 (4.89)		32.81 (3.71)		20.75 (4.43)	
Retired	252 (50.5)	9.54 (5.04)		33.20 (3.53)		21.63 (4.70)	
Other	93 (18.6)	7.05 (4.69)		33.14 (3.48)		20.53 (4.50)	
Monthly per capita income			<0.001		0.320		0.113
<5,000	256 (51.3)	8.08 (4.97)		32.99 (3.63)		21.05 (4.56)	
5,000–10,000	156 (31.3)	9.88 (4.84)		33.58 (3.35)		21.93 (4.42)	
>10,000	87 (17.4)	10.30 (5.11)		33.07 (3.64)		21.63 (4.38)	
Marital status			0.036		0.968		0.393
Married	438 (87.8)	9.14 (4.93)		33.21 (3.50)		21.52 (4.48)	
Unmarried	24 (4.8)	9.96 (5.39)		33.33 (3.34)		20.42 (4.84)	
Divorced, widowed and other situations	37 (7.4)	7.19 (5.78)		32.84 (4.22)		20.97 (4.39)	
Daily caregiver			0.075		0.224		0.470
Able to take care of themselves	92 (18.5)	9.76 (4.50)		33.04 (3.31)		21.55 (4.80)	
Parents/spouse/children and other relatives	271 (54.4)	9.10 (5.03)		33.44 (3.62)		21.53 (4.55)	
Nanny/caregiver, etc.	135 (27.1)	8.39 (5.38)		32.80 (3.56)		21.17 (4.17)	
Duration of stroke			0.541		0.013		0.172
Within 1 year	301 (60.3)	8.87 (4.91)		33.52 (3.51)		21.71 (4.65)	
1–3 years	159 (31.9)	9.35 (5.19)		32.52 (3.50)		21.06 (4.21)	
over 3 years	39 (7.8)	8.97 (5.46)		33.36 (3.74)		20.77 (4.34)	
Exercise habits before the onset of the disease			<0.001		0.002		<0.001
Yes	226 (45.3)	10.67 (5.11)		33.77 (3.31)		22.37 (4.46)	
No	273 (54.7)	7.67 (4.56)		32.71 (3.67)		20.65 (4.38)	
Positive and optimistic before the onset of the disease			0.087		0.005		0.046
Yes	448 (89.8)	9.17 (5.05)		33.35 (3.39)		21.59 (4.35)	
No	51 (10.2)	7.78 (4.81)		31.76 (4.50)		19.98 (5.45)	

The distribution of knowledge dimension revealed that the question with the highest number of participants choosing the “Very familiar” option was “Smoking, drinking, high blood pressure, diabetes, coronary heart disease, dyslipidemia, atrial fibrillation, etc. may induce or worsen stroke.” (K2), with 31.7%. The question with the highest number of participants choosing the “Have heard about it” option was “For stroke patients with mild depression/anxiety and other complications, the medical staff’s psychological rehabilitation program (including reasonable short-term exercise during the day, light music before bed to help sleep, deep breathing training, etc.) can effectively improve the patient’s quality of life.” (K9), with 55.5%. The question with the highest number of participants choosing the “Unclear” option was “Aquatic exercise therapy (i.e., hydrotherapy exercise) can effectively improve the physical function and activity participation ability of stroke patients.” (K7), with 45.9% (Supplementary Table S2).

Responses to the attitude dimension showed that 34.1% fully trusted the therapeutic, exercise, and psychological interventions provided to them by their doctors and caregivers, and were able to strictly implement them (A3), and 53.5% believed that they could adhere to the recommended programs in the long term (A7). When it comes to whether they were interested in learning about the relevant knowledge (A1), 23.6% were neutral. It is noteworthy that 18.6% strongly agreed and 31.9% agreed that their family members no longer value their opinions after the disease, that their presence in the family is diminishing, and that they are often discriminated against by other people on the road (A9) (Supplementary Table S3).

Responses to the practice dimension showed that 34.7% do not necessarily communicate the correct exercise rehabilitation program to their friends and relatives and strictly implement it with them (P3), 32.9% do not necessarily seek help from doctors/nursing staff when their psychological state is not good (P5), 19.4% seldom learn the relevant knowledge (P1), and 15.2% seldom give psychological support treatment program to their friends and relatives when they find the same psychological condition (P6) (Supplementary Table S4). When it comes to sources of knowledge about stroke and related exercise intervention and psychological intervention programs (P7), doctors and caregivers were reported by 89% of participants, followed by real-life cases around them (46.5%) (Supplementary Table S5).

In the correlation analysis, significant positive correlations were found between knowledge and attitude (*r* = 0.308, *p* = 0.002), knowledge and practice (*r* = 0.497, *p* < 0.001), and attitude and practice (*r* = 0.502, *p* < 0.001), respectively (Supplementary Table S6).

The number of participants above the cut-off value of knowledge, attitude, and practice score were 273 (54.7%), 272 (54.5%), and 255 (51.1%), respectively (Supplementary Table S7). Multivariate logistic regression showed that being college degree and above (OR = 3.36, 95% CI: [1.82, 6.22], *p* < 0.001), retired (OR = 2.22, 95% CI: [1.27, 3.90], *p* = 0.005), unmarried (OR = 3.79, 95% CI: [1.06, 13.64], *p* = 0.041), able to take care of themselves (OR = 2.47, 95% CI: [1.31, 4.65], *p* = 0.005), and without exercise habits before the onset of the disease (OR = 0.48, 95% CI: [0.32, 0.72], *p* < 0.001) were independently associated with knowledge (Table 2). Concurrently, knowledge score (OR = 1.12, 95% CI: [1.07, 1.16], *p* < 0.001) and 1–3 years of duration of stroke (OR = 0.59, 95% CI: [0.39, 0.89], *p* = 0.012) were independently associated with attitude (Table 3). Furthermore, knowledge score (OR = 1.19, 95% CI: [1.13, 1.26], *p* < 0.001), attitude score (OR = 1.28, 95% CI: [1.19, 1.38], *p* < 0.001), employed (OR = 2.41, 95% CI: [1.15, 5.04], *p* = 0.020), retired (OR = 2.20, 95% CI: [1.20, 4.02], *p* = 0.011), and more than 3 years of duration of stroke (OR = 0.37, 95% CI: [0.16, 0.85], *p* = 0.019) were independently associated with practice (Table 4).

**Table 2:** Univariate and multivariate logistic regression analysis for knowledge.

Knowledge	Univariate analysis		Multivariate analysis	
	OR (95% CI)	*p*	OR (95% CI)	*p*
Gender				
Male				
Female	0.74 (0.51, 1.06)	0.098	0.86 (0.57, 1.30)	0.482
Age				
18–44				
45–60	0.71 (0.40, 1.23)	0.219		
>60	0.80 (0.47, 1.35)	0.404		
Residence				
Rural area				
City	2.75 (1.87, 4.06)	<0.001	1.29 (0.79, 2.11)	0.310
Suburbs	1.05 (0.57, 1.94)	0.869	0.75 (0.37, 1.51)	0.417
Education				
Junior high school and below				
High school/secondary vocational school	1.91 (1.26, 2.90)	0.003	1.49 (0.91, 2.44)	0.114
College degree and above	5.52 (3.38, 9.22)	<0.001	3.36 (1.82, 6.22)	<0.001
Work status				
Employed	3.75 (2.08, 6.86)	<0.001	1.98 (0.99, 3.94)	0.053
Unemployed	1.39 (0.70, 2.78)	0.345	1.25 (0.58, 2.69)	0.574
Retired	2.59 (1.59, 4.27)	<0.001	2.22 (1.27, 3.90)	0.005
Other				
Monthly per capita income				
<5,000				
5,000–10,000	1.90 (1.27, 2.86)	0.002	1.13 (0.71, 1.82)	0.599
>10,000	2.50 (1.51, 4.23)	<0.001	1.46 (0.79, 2.70)	0.232
Marital status				
Married	1.99 (1.01, 4.06)	0.051	1.82 (0.83, 4.00)	0.135
Unmarried	4.93 (1.65, 16.44)	0.006	3.79 (1.06, 13.64)	0.041
Divorced, widowed and other situations				
Daily caregiver				
Able to take care of themselves	2.46 (1.42, 4.34)	0.002	2.47 (1.31, 4.65)	0.005
Parents/spouse/children and other relatives	1.19 (0.78, 1.79)	0.420	1.30 (0.82, 2.06)	0.272
Nanny/caregiver, etc.				
Duration of stroke				
Within 1 year				
1–3 years	1.15 (0.78, 1.70)	0.480		
over 3 years	1.14 (0.58, 2.26)	0.702		
Exercise habits before the onset of the disease				
Yes				
No	0.40 (0.27, 0.57)	<0.001	0.48 (0.32, 0.72)	<0.001
Positive and optimistic before the onset of the disease				
Yes				
No	0.60 (0.33, 1.07)	0.088	0.83 (0.42, 1.65)	0.600

**Table 3:** Univariate and multivariate logistic regression analysis for attitudes.

Attitude	Univariate analysis		Multivariate analysis	
	OR (95% CI)	*p*	OR (95% CI)	*p*
Knowledge	1.11 (1.07, 1.16)	<0.001	1.12 (1.07, 1.16)	<0.001
Gender				
Male				
Female	1.31 (0.91, 1.89)	0.153		
Age:				
18–44				
45–60	1.05 (0.61, 1.83)	0.850		
>60	0.86 (0.51, 1.45)	0.569		
Residence				
Rural area				
City	1.57 (1.08, 2.29)	0.019	1.29 (0.81, 2.04)	0.284
Suburbs	1.18 (0.64, 2.17)	0.596	1.26 (0.66, 2.42)	0.486
Education				
Junior high school and below				
High school/secondary vocational school	1.13 (0.75, 1.71)	0.565	0.86 (0.54, 1.38)	0.540
College degree and above	1.51 (0.97, 2.36)	0.070	0.87 (0.50, 1.51)	0.617
Work status				
Employed	0.86 (0.49, 1.52)	0.613		
Unemployed	0.70 (0.35, 1.37)	0.294		
Retired	0.87 (0.54, 1.41)	0.584		
Other				
Monthly per capita income				
<5,000				
5,000–10,000	1.15 (0.77, 1.71)	0.499		
>10,000	1.35 (0.83, 2.23)	0.230		
Marital status				
Married	0.80 (0.40, 1.58)	0.531		
Unmarried	0.95 (0.34, 2.75)	0.930		
Divorced, widowed and other situations				
Daily caregiver				
Able to take care of themselves	1.01 (0.60, 1.72)	0.962		
Parents/spouse/children and other relatives	1.24 (0.82, 1.88)	0.308		
Nanny/caregiver, etc.				
Duration of stroke				
Within 1 year				
1–3 years	0.61 (0.41, 0.90)	0.013	0.59 (0.39, 0.89)	0.012
over 3 years	1.29 (0.65, 2.63)	0.477	1.35 (0.65, 2.80)	0.418
Exercise habits before the onset of the disease				
Yes				
No	0.76 (0.53, 1.08)	0.131		
Positive and optimistic before the onset of the disease				
Yes				
No	0.45 (0.25, 0.82)	0.010	0.56 (0.30, 1.06)	0.074

**Table 4:** Univariate and multivariate logistic regression analysis for practices.

Practice	Univariate analysis		Multivariate analysis	
	OR (95% CI)	*p*	OR (95% CI)	*p*
Knowledge	1.23 (1.18, 1.29)	<0.001	1.19 (1.13, 1.26)	<0.001
Attitude	1.31 (1.23, 1.40)	<0.001	1.28 (1.19, 1.38)	<0.001
Gender				
Male				
Female	1.25 (0.87, 1.80)	0.234		
Age				
18–44				
45–60	1.12 (0.65, 1.93)	0.692		
>60	0.96 (0.57, 1.61)	0.871		
Residence				
Rural area				
City	1.67 (1.15, 2.44)	0.008	0.82 (0.47, 1.42)	0.477
Suburbs	1.03 (0.55, 1.88)	0.937	1.20 (0.55, 2.61)	0.652
Education				
Junior high school and below				
High school/secondary vocational school	1.40 (0.92, 2.12)	0.112	0.97 (0.56, 1.69)	0.925
College degree and above	2.29 (1.47,3.60)	<0.001	1.21 (0.62, 2.38)	0.580
Work status				
Employed	2.79 (1.57, 5.04)	0.001	2.41 (1.15, 5.04)	0.020
Unemployed	1.58 (0.79, 3.15)	0.195	1.68 (0.74, 3.85)	0.217
Retired	2.34 (1.44, 3.88)	0.001	2.20 (1.20, 4.02)	0.011
Other				
Monthly per capita income				
<5,000				
5,000–10,000	1.45 (0.97, 2.17)	0.068	0.90 (0.54, 1.51)	0.697
>10,000	1.48 (0.91, 2.43)	0.114	1.08 (0.56, 2.09)	0.814
Marital status				
Married	1.45 (0.74, 2.90)	0.281		
Unmarried	0.79 (0.27, 2.24)	0.656		
Divorced, widowed and other situations				
Daily caregiver				
Able to take care of themselves	1.21 (0.71, 2.06)	0.481		
Parents/spouse/children and other relatives	1.24 (0.82, 1.88)	0.309		
Nanny/caregiver, etc.				
Duration of stroke				
Within 1 year				
1–3 years	0.89 (0.61, 1.31)	0.562	1.04 (0.65, 1.67)	0.877
over 3 years	0.55 (0.27, 1.08)	0.087	0.37 (0.16, 0.85)	0.019
Exercise habits before the onset of the disease				
Yes				
No	0.51 (0.36, 0.73)	<0.001	0.91 (0.59, 1.41)	0.664
Positive and optimistic before the onset of the disease				
Yes				
No	0.70 (0.39, 1.25)	0.232		

**Figure 1:**
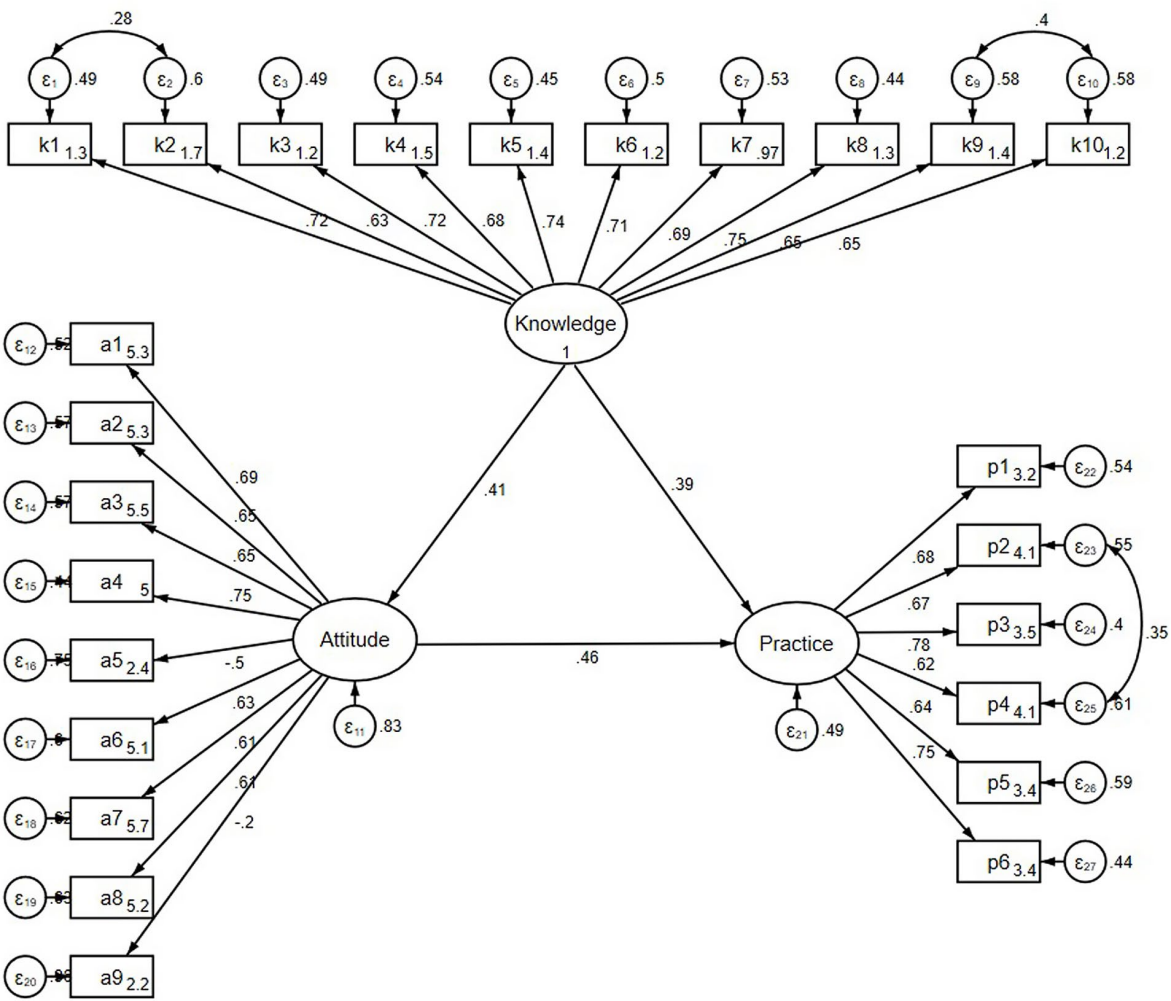
SEM model results of KAP.

The fit indices of the SEM model reached the desired range, indicating good model fit results (Supplementary Table S8) based on normal SEM model assumptions (Supplementary Table S9), the results of analysis of direct and indirect effects showed that knowledge directly affected attitude (*β* = 0.407, *p* < 0.001), knowledge (*β* = 0.390, *p* < 0.001) and attitude (*β* = 0.461, *p* < 0.001) directly affected practice, and knowledge indirectly affected practice through attitude (*β* = 0.188, *p* = 0.007) (Table 5; Figure 1).

**Table 5:** SEM model direct effect and indirect effect analysis results.

Model paths	Total effects		Direct effect		Indirect effect	
	*β* (95% CI)	*p*	*β* (95% CI)	*p*	*β* (95% CI)	*p*
Asum						
Ksum	0.407 (0.321, 0.493)	<0.001	0.407 (0.321, 0.493)	<0.001		
Psum						
Asum	0.461 (0.376, 0.546)	<0.001	0.461 (0.376, 0.546)	<0.001		
Ksum	0.578 (0.505, 0.651)	0.007	0.390 (0.304, 0.476)	<0.001	0.188 (0.136, 0.240)	0.007

## Discussion

The study indicates that while stroke patients generally exhibit a positive attitude toward home-based rehabilitation, their knowledge and practice levels remain suboptimal, highlighting a critical gap in stroke recovery programs.

Notably, the study revealed significant disparities in knowledge, attitude, and practice scores based on demographic factors such as residence, education, and prior exercise habits, suggesting that these variables play a crucial role in influencing rehabilitation outcomes. For instance, urban residents displayed higher scores in all three domains compared to their rural counterparts, a pattern that aligns with findings from other studies highlighting urban areas’ better access to health information and resources (19). These differences were substantiated by the multivariate logistic regression results, affirming the robust impact of residence on rehabilitation outcomes.

Educational attainment emerged as a critical determinant, with individuals holding a college degree or higher exhibiting superior knowledge and practice scores. This association is supported by literature indicating that higher education levels correlate with better health literacy, which in turn enhances engagement with health-promoting behaviors (20). The positive influence of employment status on rehabilitation engagement reflects the broader societal role of employment in providing structure and access to resources that facilitate health maintenance (21). Furthermore, the multivariate logistic regression results supported these findings, showing a significant prediction of higher knowledge levels by educational attainment and employment status.

Interestingly, having pre-stroke exercise habits was significantly associated with better outcomes across all KAP domains. This observation is corroborated by research suggesting that pre-existing exercise routines may enhance individuals’ resilience (22). Moreover, multivariate logistic regression reinforced these findings, indicating that factors such as being able to take care of oneself significantly predict better knowledge, which in turn influences attitudes and practices toward stroke rehabilitation.

The findings from our study using multivariate logistic regression, correlation analyses, and SEM collectively demonstrate the profound interconnectedness between KAP in the context of stroke rehabilitation, confirming that enhancements in one domain could lead to improvements in others. This is consistent with the previous study that informed patients are more likely to have positive attitudes toward their treatment options, which enhances adherence to prescribed practices (23). These findings suggest that interventions aimed at enhancing knowledge could be particularly beneficial, as increased knowledge not only uplifts attitudes but also promotes healthier practices.

The results indicate a significant variance in patients’ familiarity with various aspects of stroke, ranging from etiology to rehabilitation techniques. Notably, many patients reported being ‘unclear’ about critical rehabilitation practices such as early sports rehabilitation, aquatic exercise therapy, and specific psychological treatment programs. To address these gaps, targeted educational initiatives should be developed, emphasizing the practical application of rehabilitation techniques in everyday life. For example, utilizing popular social media platforms in China, like WeChat and Douyin, could disseminate bite-sized, easily digestible information about stroke rehabilitation. Additionally, interactive webinars hosted by healthcare professionals can offer in-depth discussions and Q&A sessions to reinforce understanding and application of the knowledge (24, 25).

The attitudes of stroke patients toward their recovery process show a generally positive inclination, but concerns about the effectiveness of long-term adherence to rehabilitation programs and fear of relapse indicate areas needing attention. Similar to previous findings, where patients’ confidence in rehabilitation positively correlated with their recovery outcomes, our study highlights the need for enhancing patients’ trust and commitment to prescribed interventions (26). To improve this, regular motivational interviewing sessions could be integrated into routine care, where healthcare providers reinforce the benefits of sustained rehabilitation efforts and address any misconceptions. Furthermore, creating patient-led support groups, particularly on platforms, where experiences and tips can be shared, may strengthen patients’ resolve and normalize their experiences, enhancing their attitudes toward ongoing care (27, 28).

In practice, while some patients regularly engage with their rehabilitation protocols, a significant number report not adhering strictly to the prescribed activities, especially in terms of sharing and implementing exercise and psychological plans with others. This reflects previous findings, which emphasized the role of social support in improving rehabilitation adherence (29). To enhance practical application, hospitals could partner with community centers to organize regular, supervised exercise sessions that also serve social functions, encouraging communal participation. Additionally, leveraging technology, such as developing a dedicated app that offers step-by-step guidance and tracking for rehabilitation exercises and psychological practices, could help patients maintain regular practice. Promotional campaigns through WeChat, where success stories and educational content are shared, could also increase engagement and compliance (30, 31).

This study has several limitations that warrant mention. First, the cross-sectional design limits our ability to establish causality between the observed knowledge, attitudes, and practices and patient outcomes, suggesting that longitudinal studies are needed to track changes over time. Second, the data were collected from a single institution, which may limit the generalizability of the findings to other settings or populations. Lastly, the reliance on self-reported measures for assessing knowledge, attitudes, and practices may introduce response biases, as participants could overestimate their compliance or understanding of rehabilitation protocols.

## Conclusion

In conclusion, stroke patients exhibit a notable disparity between their generally positive attitudes and the sufficient practices toward home-based exercise and psychological rehabilitation, despite their relatively insufficient knowledge on the subject. To enhance the effectiveness of stroke rehabilitation, healthcare providers should focus on educational interventions that increase knowledge, as this is strongly correlated with improved attitudes and practices among patients.

## Data Availability

The original contributions presented in the study are included in the article/Supplementary material, further inquiries can be directed to the corresponding authors.

## References

[1] Wang W Jiang B Sun H Ru X Sun D Wang L. Prevalence, incidence, and mortality of stroke in China: results from a nationwide population-based survey of 480 687 adults. Circulation. (2017) 135:759–71. doi: 10.1161/CIRCULATIONAHA.116.025250, PMID: 28052979

[2] GBD 2016 Stroke Collaborators. Global, regional, and national burden of stroke, 1990-2016: a systematic analysis for the global burden of disease study 2016. Lancet Neurol. (2019) 18:439–58. doi: 10.1016/S1474-4422(19)30034-1, PMID: 30871944 PMC6494974

[3] Wu S Wu B Liu M Chen Z Wang W Anderson CS. Stroke in China: advances and challenges in epidemiology, prevention, and management. Lancet Neurol. (2019) 18:394–405. doi: 10.1016/S1474-4422(18)30500-3, PMID: 30878104

[4] Zhou M Wang H Zeng X Yin P Zhu J Chen W. Mortality, morbidity, and risk factors in China and its provinces, 1990-2017: a systematic analysis for the global burden of disease study 2017. Lancet. (2019) 394:1145–58. doi: 10.1016/S0140-6736(19)30427-1, PMID: 31248666 PMC6891889

[5] Guo L Wang J Wu Q Li X Zhang B Zhou L. Clinical study of a wearable remote rehabilitation training system for patients with stroke: randomized controlled pilot trial. JMIR Mhealth Uhealth. (2023) 11:e40416. doi: 10.2196/40416, PMID: 36821348 PMC9999258

[6] Choi S Kim D. Effects of combining cognitive behavioral therapy with bilateral upper limb training in stroke patients: a randomized controlled trial. Occup Ther Int. (2022) 2022:1–9. doi: 10.1155/2022/4688113, PMID: 35912312 PMC9282985

[7] Szelenberger R Kostka J Saluk-Bijak J Miller E. Pharmacological interventions and rehabilitation approach for enhancing brain self-repair and stroke recovery. Curr Neuropharmacol. (2020) 18:51–64. doi: 10.2174/1570159X17666190726104139, PMID: 31362657 PMC7327936

[8] Walters R Collier JM Braighi Carvalho L Langhorne P Katijjahbe MA Tan D. Exploring post acute rehabilitation service use and outcomes for working age stroke survivors (≤65 years) in Australia, UK and South East Asia: data from the international AVERT trial. BMJ Open. (2020) 10:e035850. doi: 10.1136/bmjopen-2019-035850, PMID: 32532772 PMC7295421

[9] Aerts C Revilla M Duval L Paaijmans K Chandrabose J Cox H. Understanding the role of disease knowledge and risk perception in shaping preventive behavior for selected vector-borne diseases in Guyana. PLoS Negl Trop Dis. (2020) 14:e0008149. doi: 10.1371/journal.pntd.0008149, PMID: 32251455 PMC7170267

[10] Liao L Feng H Jiao J Zhao Y Ning H. Nursing assistants' knowledge, attitudes and training needs regarding urinary incontinence in nursing homes: a mixed-methods study. BMC Geriatr. (2023) 23:39. doi: 10.1186/s12877-023-03762-z, PMID: 36683023 PMC9867858

[11] Mumena WA. Maternal knowledge, attitude and practices toward free sugar and the associations with free sugar intake in children. Nutrients. (2021) 13:4403. doi: 10.3390/nu13124403, PMID: 34959955 PMC8706702

[12] Farpour H Mashhadiagha A Edrisi F Farpour S. Knowledge, attitude, and practice regarding stroke potential complications among stroke survivors' family members in shiraz, Iran. Turk J Phys Med Rehabil. (2023) 69:83–8. doi: 10.5606/tftrd.2022.9512, PMID: 37201008 PMC10186017

[13] Billinger SA Arena R Bernhardt J Eng JJ Franklin BA Johnson CM. Physical activity and exercise recommendations for stroke survivors: a statement for healthcare professionals from the American Heart Association/American Stroke Association. Stroke. (2014) 45:2532–53. doi: 10.1161/STR.0000000000000022, PMID: 24846875

[14] Gittler M Davis AM. Guidelines for adult stroke rehabilitation and recovery. JAMA. (2018) 319:820–1. doi: 10.1001/jama.2017.22036, PMID: 29486016

[15] Winstein CJ Stein J Arena R Bates B Cherney LR Cramer SC. Guidelines for adult stroke rehabilitation and recovery: a guideline for healthcare professionals from the American Heart Association/American Stroke Association. Stroke. (2016) 47:e98–e169. doi: 10.1161/STR.0000000000000098, PMID: 27145936

[16] Zhang T Zhao J Li X Bai Y Wang B Qu Y. Chinese Stroke Association guidelines for clinical management of cerebrovascular disorders: executive summary and 2019 update of clinical management of stroke rehabilitation. Stroke Vasc Neurol. (2020) 5:250–9. doi: 10.1136/svn-2019-000321, PMID: 32595138 PMC7548515

[17] He J Yang W He Q Tang Y Wang Y Wang G. Chinese pregnant women's knowledge, attitude, and practice of self-protection against coronavirus disease 2019 during the post-pandemic period: a structural equation modeling-based survey. Int J Disaster Risk Reduct. (2023) 87:103559. doi: 10.1016/j.ijdrr.2023.103559, PMID: 36714184 PMC9869621

[18] Charan J Biswas T. How to calculate sample size for different study designs in medical research? Indian J Psychol Med. (2013) 35:121–6. doi: 10.4103/0253-7176.116232, PMID: 24049221 PMC3775042

[19] Zhang QN Lu HX. Knowledge, attitude, practice and factors that influence the awareness of college students with regards to breast cancer. World J Clin Cases. (2022) 10:538–46. doi: 10.12998/wjcc.v10.i2.538, PMID: 35097079 PMC8771386

[20] Negesa LB Magarey J Rasmussen P Hendriks JML. Patients' knowledge on cardiovascular risk factors and associated lifestyle behaviour in Ethiopia in 2018: a cross-sectional study. PLoS One. (2020) 15:e0234198. doi: 10.1371/journal.pone.0234198, PMID: 32497079 PMC7271995

[21] Yuldashev M Khalikov U Nasriddinov F Ismailova N Kuldasheva Z Ahmad M. Impact of foreign direct investment on income inequality: evidence from selected Asian economies. PLoS One. (2023) 18:e0281870. doi: 10.1371/journal.pone.0281870, PMID: 36791138 PMC9931145

[22] Apóstolo J Dixe MDA Bobrowicz-Campos E Areosa T Santos-Rocha R Braúna M. Effectiveness of a combined intervention on psychological and physical capacities of frail older adults: a cluster randomized controlled trial. Int J Environ Res Public Health. (2019) 16:3125. doi: 10.3390/ijerph16173125, PMID: 31466229 PMC6747215

[23] Biyazin T Taye A Belay Y. Patient satisfaction with surgical informed consent at Jimma medical center, Ethiopia. BMC Med Ethics. (2022) 23:103. doi: 10.1186/s12910-022-00841-5 36284338 PMC9594918

[24] Ahn AB Kulhari S Karimi A Sundararajan S Sajatovic M. Readability of patient education material in stroke: a systematic literature review. Top Stroke Rehabil. (2024) 31:345–60. doi: 10.1080/10749357.2023.2259177, PMID: 37724783

[25] Szmuda T Alkhater A Albrahim M Alquraya E Ali S Dunquwah RA. YouTube as a source of patient information for stroke: a content-quality and an audience engagement analysis. J Stroke Cerebrovasc Dis. (2020) 29:105065. doi: 10.1016/j.jstrokecerebrovasdis.2020.105065, PMID: 32807469

[26] Gustavsson C von Koch L. Pain self-management intervention supports successful attainment of self-selected rehabilitation goals-secondary analysis of a randomized controlled trial. Health Expect. (2022) 25:1157–67. doi: 10.1111/hex.13469, PMID: 35285115 PMC9122423

[27] Allegue DR Kairy D Higgins J Archambault PS Michaud F Miller WC. A personalized home-based rehabilitation program using Exergames combined with a Telerehabilitation app in a chronic stroke survivor: mixed methods case study. JMIR Serious Games. (2021) 9:e26153. doi: 10.2196/26153, PMID: 34132649 PMC8441601

[28] Patel K Auton MF Watkins CL Sutton CJ Benedetto V Hackett ML. Delivering motivational interviewing early post stroke: standardisation of the intervention. Disabil Rehabil. (2022) 44:3453–8. doi: 10.1080/09638288.2020.1864035, PMID: 33355028

[29] Choukou MA Olatoye F Urbanowski R Caon M Monnin C. Digital health technology to support health care professionals and family caregivers caring for patients with cognitive impairment: scoping review. JMIR Ment Health. (2023) 10:e40330. doi: 10.2196/40330, PMID: 36630174 PMC9878361

[30] Jin Z Zhu L Zhou S Lu C. Managing post-stroke fatigue using a Mobile health called iHealth after intracerebral hemorrhage. J Multidiscip Healthc. (2024) 17:2389–97. doi: 10.2147/JMDH.S465902, PMID: 38770170 PMC11104438

[31] Kumar A Khurana D Pattanaik S Kumar M Kaur S Krishnan NC. A mobile application-based post-stroke care strategy for survivors and their caregivers for prevention and management of post-stroke complications - "stroke home care:" development and feasibility. J Neurosci Rural Pract. (2024) 15:217–26. doi: 10.25259/JNRP_411_2023, PMID: 38746514 PMC11090587

