# Supplementary material for: Stroke patients’ knowledge, attitudes, and practices regarding home-based exercise and psychological rehabilitation programs
Source: Front Med (Lausanne). 2025 Jun 26;12:1598489. doi: 10.3389/fmed.2025.1598489 (PMC12243871; doi:10.3389/fmed.2025.1598489)
Supplement: Supplementary file 2 [file Table_2.docx]

**Table S2. Knowledge dimension**

| **Knowledge** | **N（%）** | | |
| --- | --- | --- | --- |
|  | **Very familiar** | **Have heard about it** | **Unclear** |
| **1. Ischemic stroke is a clinical syndrome in which brain blood supply disorders caused by various cerebrovascular lesions lead to ischemia and hypoxic necrosis of local brain tissue, and the rapid emergence of corresponding neurological deficits.** | 91(18.2%) | 270(54.1%) | 138(27.7%) |
| **2. Smoking, drinking, high blood pressure, diabetes, coronary heart disease, dyslipidemia, atrial fibrillation, etc. may induce or worsen stroke.** | 158(31.7%) | 257(51.5%) | 84(16.8%) |
| **3. After stroke, cognitive dysfunction, depression, etc. are common complications.** | 87(17.4%) | 245(49.1%) | 167(33.5%) |
| **4. Appropriate drug intervention (such as Western antithrombotic and anticoagulant drugs) and traditional Chinese medicine nursing methods (such as traditional Chinese medicine, moxibustion, massage, etc.) can effectively control the progression and recurrence of stroke.** | 114(22.8%) | 271(54.3%) | 114(22.8%) |
| **5. Appropriate physical activity (4 times/week; 10 minutes/time of moderate-intensity aerobic activity) can effectively reduce the risk of stroke recurrence.** | 113(22.6%) | 251(50.3%) | 135(27.1%) |
| **6. Early sports rehabilitation programs need to be formulated based on functional assessment results.** | 91(18.2%) | 243(48.7%) | 165(33.1%) |
| **7. Aquatic exercise therapy (i.e. hydrotherapy exercise) can effectively improve the physical function and activity participation ability of stroke patients.** | 59(11.8%) | 211(42.3%) | 229(45.9%) |
| **8. Appropriate psychological treatment programs (including supportive care, cognitive behavioral therapy, family therapy, meditation, relaxation training, etc.) can significantly improve depression/anxiety and other related symptoms after stroke.** | 98(19.6%) | 245(49.1%) | 156(31.3%) |
| **9. For stroke patients with mild depression/anxiety and other complications, the medical staff's psychological rehabilitation program (including reasonable short-term exercise during the day, light music before bed to help sleep, deep breathing training, etc.) can effectively improve the patient's quality of life.** | 94(18.8%) | 277(55.5%) | 128(25.7%) |
| **10. For stroke patients with moderate to severe depression/anxiety and other complications, in addition to psychological rehabilitation programs, they also need to be treated with antidepressant/anti-anxiety drugs.** | 92(18.4%) | 242(48.5%) | 165(33.1%) |
