# Supplementary material for: Stroke patients’ knowledge, attitudes, and practices regarding home-based exercise and psychological rehabilitation programs
Source: Front Med (Lausanne). 2025 Jun 26;12:1598489. doi: 10.3389/fmed.2025.1598489 (PMC12243871; doi:10.3389/fmed.2025.1598489)
Supplement: Supplementary file 3 [file Table_3.docx]

**Table S3. Attitude dimension**

| **Attitude** | **Strongly agree** | **Agree** | **Neutral** | **Disagree** | **Strongly disagree** |
| --- | --- | --- | --- | --- | --- |
| **1. You are very interested in learning the causes, treatment methods, exercise intervention programs, psychological intervention programs and other related knowledge of stroke.** | 107(21.4%) | 263(52.7%) | 118(23.6%) | 10(2%) | 1(0.2%) |
| **2. You believe that stroke is a serious disease that requires you and your caregivers to pay attention to details.** | 167(33.5%) | 244(48.9%) | 75(15%) | 9(1.8%) | 4(0.8%) |
| **3. You trust the treatment plans, exercise and psychological intervention plans provided to you by doctors and nursing staff, and confirm that you can strictly implement the exercise and psychological rehabilitation plans provided by doctors and therapists.** | 170(34.1%) | 250(50.1%) | 68(13.6%) | 8(1.6%) | 3(0.6%) |
| **4. You know the key risk factors for disease progression and pay careful attention to these details in your daily life.** | 132(26.5%) | 243(48.7%) | 108(21.6%) | 13(2.6%) | 3(0.6%) |
| **5. You are worried about the possibility of relapse even if you strictly follow the exercise and psychological rehabilitation programs given by doctors and caregivers.** | 122(24.4%) | 236(47.3%) | 101(20.2%) | 36(7.2%) | 4(0.8%) |
| **6. Do you believe that home-based rehabilitation exercise training and psychological rehabilitation programs are necessary to prevent disease progression and relapse.** | 139(27.9%) | 241(48.3%) | 105(21%) | 12(2.4%) | 2(0.4%) |
| **7. Do you think you can continue to follow the exercise and psychological rehabilitation programs recommended to you by doctors and nursing staff for a long time?** | 147(29.5%) | 267(53.5%) | 75(15%) | 10(2%) | 0 (0%) |
| **8. Do you think you should increase communication with your family and medical staff when you are depressed?** | 165(33.1%) | 247(49.5%) | 73(14.6%) | 10(2%) | 4(0.8%) |
| **9. Do you think that after getting sick, your family will no longer value your opinions and your presence in the family will gradually decrease; and you will often be discriminated against by others on the road.** | 93(18.6%) | 159(31.9%) | 112(22.4%) | 105(21%) | 30(6%) |
