# Supplementary material for: Stroke patients’ knowledge, attitudes, and practices regarding home-based exercise and psychological rehabilitation programs
Source: Front Med (Lausanne). 2025 Jun 26;12:1598489. doi: 10.3389/fmed.2025.1598489 (PMC12243871; doi:10.3389/fmed.2025.1598489)
Supplement: Supplementary file 4 [file Table_4.docx]

**Table S4. Practice dimension (single choice questions)**

| **Practice** | **Completely meets** | **Meets** | **Not necessarily** | **Does not meet** | **Completely does not meet** |
| --- | --- | --- | --- | --- | --- |
| **1. You will often learn about the causes of stroke, treatment methods, exercise intervention programs, psychological intervention programs and other related knowledge.** | 60(12%) | 167(33.5%) | 157(31.5%) | 97(19.4%) | 18(3.6%) |
| **2. You have been insisting on implementing the sports rehabilitation plan and psychological treatment plan given by the doctor/nursing staff.** | 123(24.6%) | 228(45.7%) | 100(20%) | 42(8.4%) | 6(1.2%) |
| **3. You will often pass on the correct sports rehabilitation program to your surrounding relatives and friends, and strictly implement it with them.** | 77(15.4%) | 174(34.9%) | 173(34.7%) | 59(11.8%) | 16(3.2%) |
| **4. When you implement the exercise program, your process and results will be supervised by relatives or medical/nursing staff.** | 142(28.5%) | 193(38.7%) | 119(23.8%) | 41(8.2%) | 3(0.6%) |
| **5. When you find yourself in a poor mental state, you will habitually seek help from doctors/nursing staff.** | 95(19%) | 158(31.7%) | 164(32.9%) | 69(13.8%) | 13(2.6%) |
| **6. When you find that your similarly sick relatives and friends around you have psychological problems, you will promptly provide them with psychological support and treatment plans.** | 75(15%) | 181(36.3%) | 153(30.7%) | 76(15.2%) | 14(2.8%) |
