# Supplementary material for: Stroke patients’ knowledge, attitudes, and practices regarding home-based exercise and psychological rehabilitation programs
Source: Front Med (Lausanne). 2025 Jun 26;12:1598489. doi: 10.3389/fmed.2025.1598489 (PMC12243871; doi:10.3389/fmed.2025.1598489)
Supplement: Supplementary file 5 [file Table_5.docx]

**Table S5. Practice Dimensions (Multiple Choice Questions)**

| **Practice** | **Select this option** | |
| --- | --- | --- |
| **7. From what channels do you generally learn about stroke and related exercise intervention and psychological intervention programs:** | |  |
| Relevant books | | 115(23%) |
| Doctors and nursing staff | | 444(89%) |
| Internet media | | 167(33.5%) |
| Real-life cases around you | | 232(46.5%) |
| Other methods | | 74(14.8%) |
