# Supplementary material for: Stroke patients’ knowledge, attitudes, and practices regarding home-based exercise and psychological rehabilitation programs
Source: Front Med (Lausanne). 2025 Jun 26;12:1598489. doi: 10.3389/fmed.2025.1598489 (PMC12243871; doi:10.3389/fmed.2025.1598489)
Supplement: Supplementary file 6 [file Table_6.docx]

**Table S6. Correlation analysis**

|  | **Knowledge** | **Attitude** | **Practice** |
| --- | --- | --- | --- |
| **Knowledge** | 1.000 |  |  |
| **Attitude** | 0.308 (P<0.001) | 1.000 |  |
| **Practice** | 0.497 (P<0.001) | 0.502 (P<0.001) | 1.000 |
