# Supplementary material for: Stroke patients’ knowledge, attitudes, and practices regarding home-based exercise and psychological rehabilitation programs
Source: Front Med (Lausanne). 2025 Jun 26;12:1598489. doi: 10.3389/fmed.2025.1598489 (PMC12243871; doi:10.3389/fmed.2025.1598489)
Supplement: Supplementary file 7 [file Table_7.docx]

**Table S7. Univariate and multivariate analysis cutoff values**

| **Cutoff values: knowledge, attitude, practice as the median** | **N (%)** |
| --- | --- |
| Total score of knowledge dimension |  |
| **Ksum>=9** | 273 (54.7%) |
| **Ksum<=8** | 226 (45.3%) |
| Attitude dimension total score |  |
| **Asum>=33** | 272 (54.5%) |
| **Asum<=32** | 227 (45.5%) |
| Total score of practice dimension |  |
| **Psum>=22** | 255 (51.1%) |
| **Psum <=21** | 244 (48.9%) |
