# Supplementary material for: Stroke patients’ knowledge, attitudes, and practices regarding home-based exercise and psychological rehabilitation programs
Source: Front Med (Lausanne). 2025 Jun 26;12:1598489. doi: 10.3389/fmed.2025.1598489 (PMC12243871; doi:10.3389/fmed.2025.1598489)
Supplement: Supplementary file 8 [file Table_8.docx]

**Table S8. SEM model fitting situation**

| **Indicators** | **Reference** | **Results** |
| --- | --- | --- |
| RMSEA | <0.08Good | 0.055 |
| SRMR | <0.08Good | 0.055 |
| TLI | >0.8Good | 0.914 |
| CFI | >0.8Good | 0.923 |
