# Supplementary material for: Stroke patients’ knowledge, attitudes, and practices regarding home-based exercise and psychological rehabilitation programs
Source: Front Med (Lausanne). 2025 Jun 26;12:1598489. doi: 10.3389/fmed.2025.1598489 (PMC12243871; doi:10.3389/fmed.2025.1598489)
Supplement: Supplementary file 9 [file Table_9.docx]

**Table S9.** **SEM model assumptions**

| **Indicators** |  | **Estimate** | **P>\|z\|** |
| --- | --- | --- | --- |
| Asum |  |  |  |
|  | Ksum | 9.25 | <0.001 |
| Psum |  |  |  |
|  | Asum | 10.62 | <0.001 |
|  | Ksum | 15.56 | 0.007 |
