# Supplementary material for: Stroke patients’ knowledge, attitudes, and practices regarding home-based exercise and psychological rehabilitation programs
Source: Front Med (Lausanne). 2025 Jun 26;12:1598489. doi: 10.3389/fmed.2025.1598489 (PMC12243871; doi:10.3389/fmed.2025.1598489)
Supplement: Supplementary file 10 [file Table_10.doc]

**Stroke Patients' Knowledge, Attitudes, and Practices Regarding Home-Based Exercise and Psychological Rehabilitation Programs**

| **Part 1: Basic Information** | | | |  |
| --- | --- | --- | --- | --- |
| **1.Your gender:** | | 1. Male | 1. Female |  |
| **2. Your age:** | | | |  |
| **3. Your place of residence:** | | 1. Rural area 2. City 3. Suburbs | |  |
| **4.Your education level:** | | 1. Junior high school and below 2. High school/technical secondary school 3. College/Undergraduate 4. Master degree and above | |  |
| **5.Your work status:** | | 1. On the job 2. Unemployed 3. retire 4. other | |  |
| **6. In the past year, your family’s monthly per capita income was (including physical income and rental income, etc.): ______ yuan** | | 1. <2000 2. 2000-5000 3. 5000-10000 4. 10000-20000 5. >20000 | |  |
| **7.Your marital status:** | | 1. Married 2. Unmarried 3. Divorced, widowed and other situations | |  |
| **8. Your daily caregiver is:** | | 1. Able to take care of themselves 2. Relatives such as parents/spouse/children 3. Nanny/caregiver 4. Other | |  |
| **9. You have had stroke since:** | | 1. Within 1 year 2. 1-3 years 3. Over 3 years | |  |
| **10. Did you have exercise habits before the onset of the disease:** | | 1. Yes 2. No | |  |
| **11. Were you a positive and optimistic person before the onset of the disease (not in a depressed state):** | 1. Yes 2. No | | | |
| **12.Do you have a stroke:** | 1. Yes 2. No | | | |

| **Part 2: Stroke patients’ understanding of home exercise and psychological rehabilitation programs to improve physical, mental and depression status** | | | |  |
| --- | --- | --- | --- | --- |
| **To what extent do you understand the following statements:** | | | | |
| 1. Ischemic stroke is a clinical syndrome in which brain blood supply disorders caused by various cerebrovascular lesions lead to ischemia and anoxic necrosis of local brain tissue, and the rapid emergence of corresponding neurological deficits; | a. Very familiar | b. Partly familiar | c.Unclear | |
| 1. Smoking, drinking, high blood pressure, diabetes, coronary heart disease, dyslipidemia, atrial fibrillation, etc. may induce or worsen stroke; | a. Very familiar | b. Partly familiar | c.Unclear | |
| 1. After stroke, cognitive dysfunction, depression, etc. are common complications; | a. Very familiar | b. Partly familiar | c.Unclear | |
| 1. Appropriate drug intervention (such as Western antithrombotic and anticoagulant drugs) and traditional Chinese medicine nursing methods (such as traditional Chinese medicine, moxibustion, massage, etc.) can effectively control the progression and recurrence of stroke; | a. Very familiar | b. Partly familiar | c.Unclear | |
| 1. Appropriate physical activity (4 times/week; 10 minutes/time of moderate-intensity aerobic activity) can effectively reduce the risk of stroke recurrence; | a. Very familiar | b. Partly familiar | c.Unclear | |
| 1. Early motor rehabilitation programs need to be formulated based on functional assessment results; | a. Very familiar | b. Partly familiar | c.Unclear | |
| 1. Aquatic exercise therapy (i.e. hydrotherapy) can effectively improve the physical function and activity participation ability of stroke patients; | a. Very familiar | b. Partly familiar | c.Unclear | |
| 1. Appropriate psychological treatment programs (including supportive care, cognitive behavioral therapy, family therapy, meditation, relaxation training, etc.) can significantly improve depression/anxiety and other related symptoms after stroke; | a. Very familiar | b. Partly familiar | c.Unclear | |
| 1. For stroke patients with mild depression/anxiety and other complications, the medical staff's psychological rehabilitation program (including reasonable short-term exercise during the day, light music before bed to help sleep, deep breathing training, etc.) can effectively improve the patient's quality of life; | a. Very familiar | b. Partly familiar | c.Unclear | |
| 1. For stroke patients with moderate to severe depression/anxiety and other complications, in addition to psychological rehabilitation programs, they also need to be treated with antidepressant/anti-anxiety drugs; | a. Very familiar | b. Partly familiar | c.Unclear | |

| **Part 3: Stroke patients’ attitudes towards home exercise and psychological rehabilitation programs to improve their physical, mental and depression status** | | | | | |
| --- | --- | --- | --- | --- | --- |
| 1. You are very interested in learning the causes, treatment methods, exercise intervention programs, psychological intervention programs and other related knowledge of stroke (P); | a. Strongly agree | b.Agree | c. Neutrality | d. Disagree | e.Strongly disagree |
| 1. You believe that stroke is a serious disease that requires you and your caregivers to pay attention to details (P); | a. Strongly agree | b.Agree | c. Neutrality | d. Disagree | e.Strongly disagree |
| 1. You trust the treatment plans and exercise and psychological intervention plans provided to you by doctors and caregivers, and confirm that you can strictly implement the exercise and psychological rehabilitation plans provided by doctors and therapists (P); | a. Strongly agree | b.Agree | c. Neutrality | d. Disagree | e.Strongly disagree |
| 1. You are aware of key risk factors related to disease progression and pay great attention to these details in your daily life (P); | a. Strongly agree | b.Agree | c. Neutrality | d. Disagree | e.Strongly disagree |
| 1. You are worried about the possibility of relapse even if you strictly follow the exercise and psychological rehabilitation programs given by doctors and caregivers (N); | a. Strongly agree | b.Agree | c. Neutrality | d. Disagree | e.Strongly disagree |
| 1. Do you believe that home rehabilitation exercise training and psychological rehabilitation programs are necessary to prevent disease progression and recurrence (P); | a. Strongly agree | b.Agree | c. Neutrality | d. Disagree | e.Strongly disagree |
| 1. Do you think you can persist in following the exercise and psychological rehabilitation programs recommended to you by doctors and nursing staff for a long time (P); | a. Strongly agree | b.Agree | c. Neutrality | d. Disagree | e.Strongly disagree |
| 1. Do you think you should increase communication with family members and medical staff when you are feeling depressed (P); | a. Strongly agree | b.Agree | c. Neutrality | d. Disagree | e.Strongly disagree |
| 1. You feel that your personality has changed after getting sick, which has also affected your social life, and you are more worried that your disease will become a burden to your family (N); | a. Strongly agree | b.Agree | c. Neutrality | d. Disagree | e.Strongly disagree |
| 1. Do you think that after getting sick, your family will no longer value your opinions and your presence in the family will gradually decrease; and you will often be discriminated against by others on the road (N); | a. Strongly agree | b.Agree | c. Neutrality | d. Disagree | e.Strongly disagree |

| **Part 4: Practice of stroke patients using home exercise and psychological rehabilitation programs to improve their physical, mental and depression status** | | | | | |
| --- | --- | --- | --- | --- | --- |
| 1. You will often learn about the causes, treatment methods, exercise intervention programs, psychological intervention programs and other related knowledge of stroke (P); | a.Always | b. Often | c.Sometimes | d. Very few | e. Never |
| 1. You have always insisted on implementing the sports rehabilitation plan and psychological treatment plan given by the doctor/nursing staff (P); | a.Always | b. Often | c.Sometimes | d. Very few | e. Never |
| 1. You will often pass on the correct sports rehabilitation program to surrounding relatives and friends, and strictly implement it with them (P); | a.Always | b. Often | c.Sometimes | d. Very few | e. Never |
| 1. When you implement the exercise program, your process and results will be supervised by relatives or medical/nursing staff (P); | a.Always | b. Often | c.Sometimes | d. Very few | e. Never |
| 1. When you find yourself in a poor mental state, you will habitually seek help from doctors/nursing staff (P); | a.Always | b. Often | c.Sometimes | d. Very few | e. Never |
| 1. When you discover that similarly ill relatives and friends around you have psychological problems, you will promptly provide them with psychological support and treatment plans (P); | a.Always | b. Often | c.Sometimes | d. Very few | e. Never |
| 1. From what channels do you Neutrality learn about stroke and related exercise intervention and psychological intervention programs (multiple choice questions); | a.Related books | b.Doctors and nursing staff | c.Online media | d.Real cases around us | e.Other ways |
